# Integrative bioinformatics approach yields a novel gene expression risk model for prognosis and progression prediction in prostate cancer

**DOI:** 10.1111/jcmm.18405

**Published:** 2024-06-06

**Authors:** Yunyan Zhang, Zhuolin Liu, Liu Yu, Aoyu Fan, Yunpeng Li, Xiaobo Li, Wei Chen

**Affiliations:** ^1^ Department of Urology Zhongshan Hospital, Fudan University Shanghai China; ^2^ School of Basic Medical Sciences Fudan University Shanghai China

**Keywords:** progression‐related genes, prostate cancer, single‐cell RNA‐seq analysis, SYK

## Abstract

Prostate cancer (PCa), a prevalent malignancy among elderly males, exhibits a notable rate of advancement, even when subjected to conventional androgen deprivation therapy or chemotherapy. An effective progression prediction model would prove invaluable in identifying patients with a higher progression risk. Using bioinformatics strategies, we integrated diverse data sets of PCa to construct a novel risk model predicated on gene expression and progression‐free survival (PFS). The accuracy of the model was assessed through validation using an independent data set. Eight genes were discerned as independent prognostic factors and included in the prediction model. Patients assigned to the high‐risk cohort demonstrated a diminished PFS, and the areas under the curve of our model in the validation set for 1‐year, 3‐year, and 5‐year PFS were 0.9325, 0.9041 and 0.9070, respectively. Additionally, through the application of single‐cell RNA sequencing to two castration‐related prostate cancer (CRPC) samples and two hormone‐related prostate cancer (HSPC) samples, we discovered that luminal cells within CRPC exhibited an elevated risk score. Subsequent molecular biology experiments corroborated our findings, illustrating heightened SYK expression levels within tumour tissues and its contribution to cancer cell migration. We found that the knockdown of SYK could inhibit migration in PCa cells. Our progression‐related risk model demonstrated the potential prognostic value of SYK and indicated its potential as a target for future diagnosis and treatment strategies in PCa management.

## 1 INTRODUCTION

Prostate cancer (PCa) stands as one of the most prevalent malignant conditions affecting elderly males. In the year 2020 alone, there were an estimated 1.4 million newly diagnosed cases of PCa worldwide, with approximately 374,000 individuals succumbing to this disease.^1^ Radical prostatectomy (RP) and radiotherapy continue to represent the prevailing treatment modalities for patients with localized PCa. Nonetheless, a notable proportion of PCa patients, ranging from 15% to 34%, encounter biochemical recurrences (BCR) within a decade following their initial diagnosis. These recurrences are typically identified by a progressive elevation in serum prostate‐specific antigen (PSA) levels, reaching at least 0.2 ng/mL.^2^ The presence of BCR after prostatectomy often indicates an unfavourable prognosis for patients. Those who experience early BCR within a 2‐year timeframe face a heightened risk of tumour progression and cancer‐specific mortality.^3^ These findings suggest that a shorter time of BCR is associated with a higher risk of castration‐related prostate cancer (CRPC) development. Detection of BCR assumes paramount importance in tailoring effective management strategies for PCa. However, the factors underlying the onset of BCR remain elusive, and the inherent heterogeneity of PCa presents challenges in accurately predicting the timing of BCR occurrence for individual patients.^4^ Currently, the traditional Gleason score (GS) is used in clinical practice to predict the prognosis of PCa. However, even patients with the same GS and similar stages often display considerable variations in their prognosis. Consequently, formulating a personalized and precise predictive approach for time‐to‐BCR remains a significant hurdle.

In clinical practice, the identification of BCR typically triggers the implementation of secondary treatments for patients with PCa following RP, including hormone therapy and salvage interventions.^5^ Among these approaches, androgen deprivation therapy (ADT) takes precedence as the primary hormone‐based option for managing metastatic or advanced PCa by curbing disease progression through the reduction of circulating testosterone levels.^6^ Initially, a substantial proportion of patients display a favourable response to ADT either as a stand‐alone treatment or in combination with docetaxel chemotherapy.^7^ Nevertheless, the relentless course of PCa persists, leading to the development of CRPC over a span of months or years. Unfortunately, CRPC represents an incurable condition, typically associated with a life expectancy of under 3 years.^8^, ^9^ Driving factors underpinning the emergence of castration resistance encompass genetic alterations such as amplifications and mutations of the androgen receptor (AR) gene, along with epigenetic regulation of AR mediated by microRNAs.^10^, ^11^, ^12^ Intriguingly, certain genetic or molecular events observed in CRPC can also manifest in primary PCa, implying that the underlying mechanisms driving the progression from RP‐treated PCa to BCR may partially overlap with those inciting the development of CRPC. While numerous biosignatures and clinical characteristics have been explored for the prediction of BCR, the available studies in this regard remain limited, with even fewer investigations delving into the latent prognostic significance of genome‐wide expression changes in CRPC.

Considering the limitations of the published evidence, in this study, we attempted to develop a robust risk model to predict the progression‐free survival (PFS) of PCa. By comparing The Cancer Genome Atlas (TCGA)‐Prostate Adenocarcinoma (PRAD) to West Coast Dream Team (WCDT)‐metastatic CRPC (mCRPC), along with the hormone‐sensitive cell line (LNCaP) contrasted against the castration‐resistant cell line (C4‐2), we embarked on a transcriptomic examination to explore the genetic alterations accompanying PCa progression. A progression‐related risk model (PRM) was further investigated using progression‐related genes to predict the PFS of patients with PCa. Subsequently, through an investigation of the PRM in single‐cell RNA‐sequencing data sets, we were able to identify that luminal cells of CRPC exhibited elevated risk scores and showed higher AR response scores. Then, we validated the expression of these candidate genes via qPCR and sought to elucidate the role of SYK in the migratory behaviour of PCa cells. Collectively, our research unveiled the strong association between the PRM and prognostic prediction in PCa patients, consequently providing novel insights into the clinical treatment and management of individuals affected by PCa.

## 2 MATERIALS AND METHODS

### 2.1 Data acquisition

In this study, the R package ‘TCGAbiolinks’ (Version 2.25.2) was used to download transcriptome profiles, proteome profiles and clinical data of TCGA‐PRAD. The transcriptome profiles of WCDT‐mCRPC were also downloaded using R software.^13^, ^14^ Stockholm (GSE70769), Taylor (GSE21034) and the German Cancer Research Center (DKFZ) (DKFZ2018) were downloaded from the Prostate Cancer Database (PCaDB).^15^ The original RNA sequencing data of LNCaP and C4‐2 cell lines (GSE180373) were downloaded from the Sequence Read Archive (SRA) (https://www.ncbi.nlm.nih.gov/sra). We used the STAR 2‐pass workflow to perform raw data mapping of GSE180373 to obtain the count matrix.^16^

Single‐cell RNA sequencing was conducted on samples obtained from patients afflicted with CRPC, as well as another duo of samples acquired from patients with hormone‐sensitive prostate cancer (HSPC). The expression count matrix was normalized by the R package ‘Seurat’ and the top 3000 genes were selected as high‐variable genes.

### 2.2 Differentially expressed gene analysis

Using R package ‘DESeq2’ (Version 1.34.0), the DEGs were screened out with baseMean > Q1, *p* < 0.01, and up/down DEGs |logFC| > mean of | logFC | as conditions. The R package ‘RobustRankAggreg’ (Version 1.1) was performed to integrate the DEGs of cells and tissues with *p* < 0.05. The volcano plot and heatmap were performed with R packages ‘EnhancedVolcano’ and ‘ComplexHeatmap’ to show DEGs. The intersection of DEGs was shown with a Venn diagram by R package ‘ggVennDiagram’. The R package ‘Hmisc’ (Version 4.7.0) was applied to analyse the correlation of genes.

### 2.3 Enrichment analysis

Gene Ontology Analysis (GO), Kyoto Encyclopedia of Genes and Genomes (KEGG)/Reactome Pathway analysis was implemented with the R packages ‘clusterProfiler’ (Version 4.5.1.902) and ‘ReactomePA’(Version 1.41.0.991). Adjusted *p* value <0.05 was considered statistically significant. The R package ‘CBNplot’ (Version 0.99.2) was used to predict upstream and downstream relationships of signalling pathways.

### 2.4 Single‐cell RNA sequencing analysis

The risk score of all cells was calculated by Nomogram. Pathway enrichment analysis was performed by iterative gene set enrichment analysis (irGSEA) using the method of single‐sample Gene Set Enrichment Analysis (ssGSEA).

### 2.5 Quantitative real‐time PCR assay

The total RNA of tissues and cells was extracted by RNAiso Plus (Takara, Beijing, China). Reverse transcription was carried out by ReverTra AceTM qPCR RT Kit (Toyobo, Shanghai, China). Real‐time qPCR was carried out by Hieff® qPCR SYBR Green Master Mix (11203ES03, Yeasen, Shanghai, China) according to the manufacturer's instructions. Beta‐actin was used as the internal reference for mRNA expression. Primer sequences are listed in Table S1.

### 2.6 Human samples

Our research was approved by the Institutional Research Ethics Committee of Zhongshan Hospital, Fudan University (Approval number: B2020‐351R) and written informed consent was obtained from all patients before surgery. All human samples were collected from patients who have gained primary PCa and undergone radical prostatectomy at Zhongshan Hospital, Fudan University in Shanghai, China. Tissue blocks were obtained from the tumour nodules. Upon careful evaluation of the histological sections stained with haematoxylin and eosin (H&E) on both sides of the tissue blocks, the lead study pathologist selected a segment of tumour specimens from the central regions of the inspected block for the following study.

### 2.7 Construction and validation of the progression‐related model to evaluate the progression‐related risk score

Four independent PCa data sets, TCGA‐PRAD (*n* = 547), DKFZ (*n* = 118), Stockholm (*n* = 94) and Taylor (*n* = 185) were integrated to create one data set incorporating patients with complete clinical data. The hazard ratio forest plot was created using the R package ‘forestplot’. The correlation of gene expression was shown using the R package ‘ggcorrplot’. Using the R package ‘caTools’ (Version 1.18.2), PCa patients from four data sets were divided into training and validation sets by stratified sampling. Ridge regression was performed with the R package ‘glmnet’ (Version 4.1.2) to obtain the coefficient that was visualized by a nomogram using the R package ‘regplot’.^17^, ^18^ The R package ‘survcomp’ was used to calculate the C‐index and 95% CI.^19^, ^20^ Using the R package ‘timeROC’, receiver operating characteristic (ROC) curves of 1 year, 3 years and 5 years were applied to assess the sensitivity and specificity of our model based on the area under the curve (AUC).^21^ We executed the R package ‘survIDINRI’ to calculate net reclassification improvement (NRI) and integrated discrimination improvement (IDI) between progression‐related risk score (PRS) and PSA level for predicting 3‐year PFS. We used the R package ‘survival’ (Version 3.3.1) to perform survival analysis. The plots were shown by the R package ‘survminer’.

### 2.8 Wound healing assay

PCa cells were cultured in six‐well plates to confluence, and wounds were created with 10 μL pipette tips. Then, cells were incubated in the serum‐free culture medium, and images were taken from four randomly selected fields at 0 h and 48 h, and the migrating rate was calculated using ImageJ (Fiji) software.

### 2.9 Cell proliferation assay

The Cell Counting Kit‐8 assay (Yeasen, Shanghai, China) was used to detect cell proliferation ability. 2000 cells were seeded in each well of a 96‐well plate. Subsequently, 10 μL CCK‐8 solution was added into the culture medium in the wells at 0, 2, 4 and 6 days after transfection of siRNA. After incubation for 2 h at 37 °C, the absorbance at 450 nm was measured by a microplate reader to reflect cell proliferation ability.

### 2.10 Cell migration assay

For the cell migration assay, PCa cells were cultured in DMEM supplemented with 0.5% FBS for 24 h.1 × 10^5^ PCa cells were seeded into the upper transwell chambers (8.0 μm) for 24 h, and then cells in the upper chambers were removed and transmembrane cells were fixed with 4% paraformaldehyde for 10 min and then stained with crystal violet for 20 min. Photographs of five random fields per membrane were taken at 100× magnification, and the stained cells were counted using ImageJ (Fiji) software.

### 2.11 Cell culture and RNA interference

Normal epithelial cell line from the prostate (RWPE‐1) and prostate cancer cell lines including DU145, PC‐3, 22RV1, C4‐2 and LNCaP were all purchased from Stem Cell Bank, Chinese Academy of Sciences, and cultured as previously described.^22^ SiRNA transfection was performed using Lipofectamine 3000 Reagent (Invitrogen, USA) according to the manufacturer's instrument. The siRNA sequences targeting SYK were as follows: siSYK‐1: 5′‐GCACTATCGCATCGACAAA‐3′; siSYK‐2: 5′‐GTCGAGCATTATTCTTATA‐3′ (Ribobio, Guangzhou, Guangdong, China).

### 2.12 Western blotting

Total protein was isolated with RIPA Lysis Buffer (Beyotime, Shanghai, China). The protein concentration was quantified using a BCA kit (Beyotime, Shanghai, China). Then, western blotting analysis was conducted as described previously.^23^ β‐actin protein was used as a loading control, and the primary antibodies included anti‐SYK (Cell Signalling Technology Cat# 2712), and anti‐GAPDH (Cell Signalling Technology Cat# 2118).

### 2.13 Statistical analysis

All statistical analyses were performed using R software (Version 4.1.3) and Graphad Prism 9.0. For comparisons between the two groups, two‐tailed Student's *t*‐test and Wilcoxon tests were conducted. For comparisons among more than two groups, one‐way ANOVA was performed. Categorical data were analysed using the chi‐square test. Pearson's correlation coefficient was used to measure the correlation between two continuous variables. Kaplan–Meier (KM) survival curves were compared using the log‐rank test. Statistical significance was set at *p* < 0.05.

## 3 RESULTS

### 3.1 Identification of progression‐related differentially expressed genes and progression‐related subgroups in PCa

**FIGURE 1:**
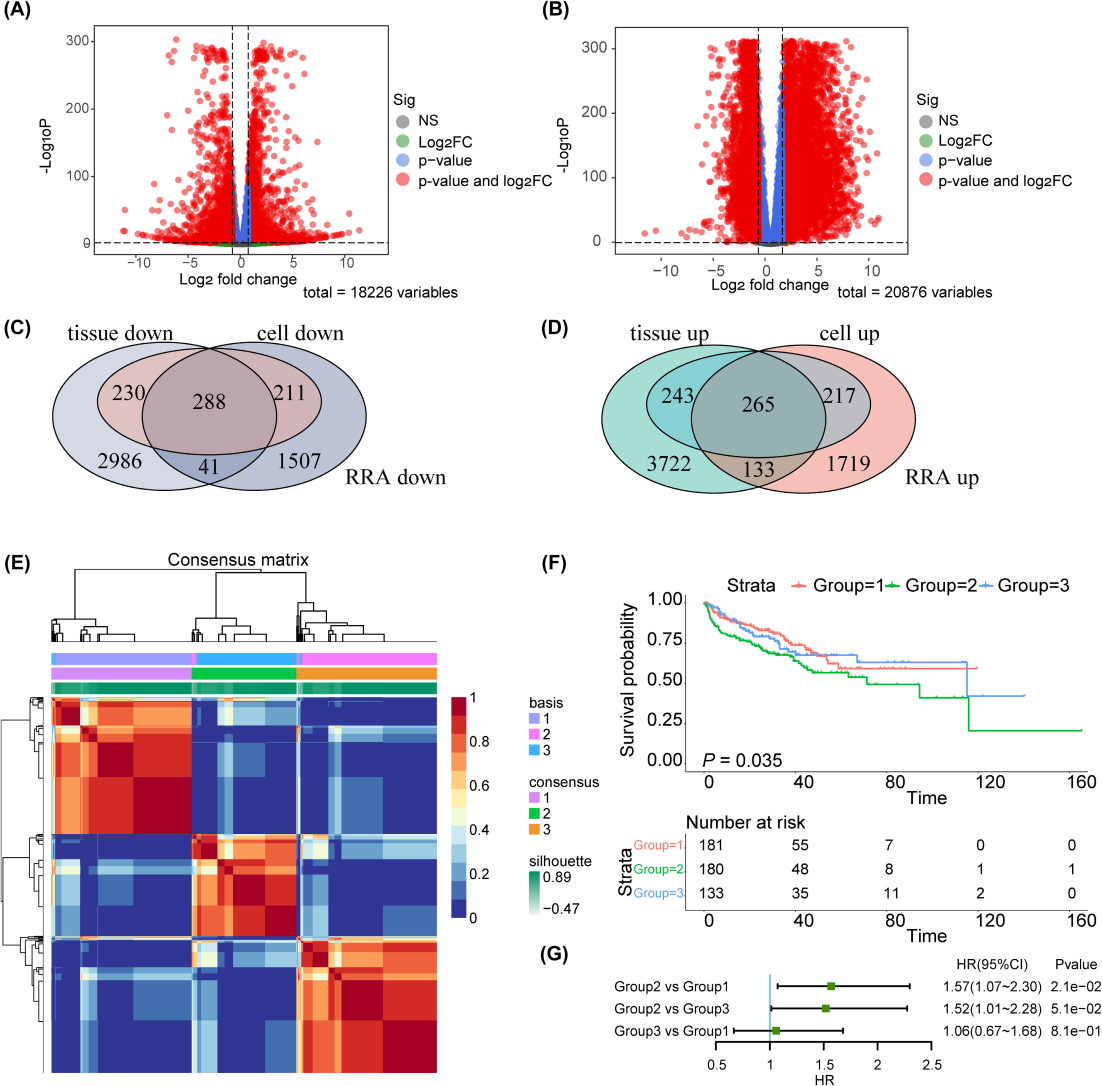
Identification of progression‐related differentially expressed genes and construction of progression‐related subgroups. (A) Volcano plot of RNA sequencing of PCa cell lines comparing C4‐2 to LNCaP. (B) Volcano plot of RNA sequencing of PCa samples comparing mCRPC to TCGA‐PRAD. (C, D) Venn diagram of consistently downregulated and upregulated genes in both comparisons. (E) Consensus matrix of PRAD patients into three different subgroups. (F) The Kaplan–Meier curves of different progression‐related subgroups. (G) Forest plot showing HR and 95% CI of different groups.

By comparing C4‐2 to LNCaP cells, a total of 8078 differentially expressed genes (DEGs) were identified, with 2334 DEGs showing upregulation and 2047 DEGs displaying downregulation (Figure 1A). Comparing CRPC tissues to the HSPC ones, 4363 genes were upregulated and 3715 genes were downregulated (Figure 1B). Using the R package ‘RobustRankAggreg’, we integrated DEGs from both comparisons and finally obtained 729 consistently downregulated DEGs and 725 consistently upregulated DEGs, which were regarded as progression‐related DEGs (prDEGs) (Figure 1C,D).

To investigate the potential association between progression‐related prDEGs and the recurrence and progression of PCa, we employed a nonnegative matrix factorization (NMF)^24^ approach to classify PCa patients from TCGA‐PRAD into distinct subgroups based on the expression patterns of prDEGs (Figure 1E, Figure S1–S4). Three distinct subgroups were discovered: 181 cases in Group 1, 180 cases in Group 2 and 133 cases in Group 3. The KM curves of the different subgroups showed a significant difference in terms of PFS (Table S2, Figure 1F). The prognosis of Group 2 patients is inferior to that of both Group 1 and Group 3 (Figure 1G). These results strongly indicated that prDEGs exert a critical role in predicting the prognosis of patients with PRAD.

### 3.2 Characteristics of prDEGs and screening of key survival‐related prDEGs

**FIGURE 2:**
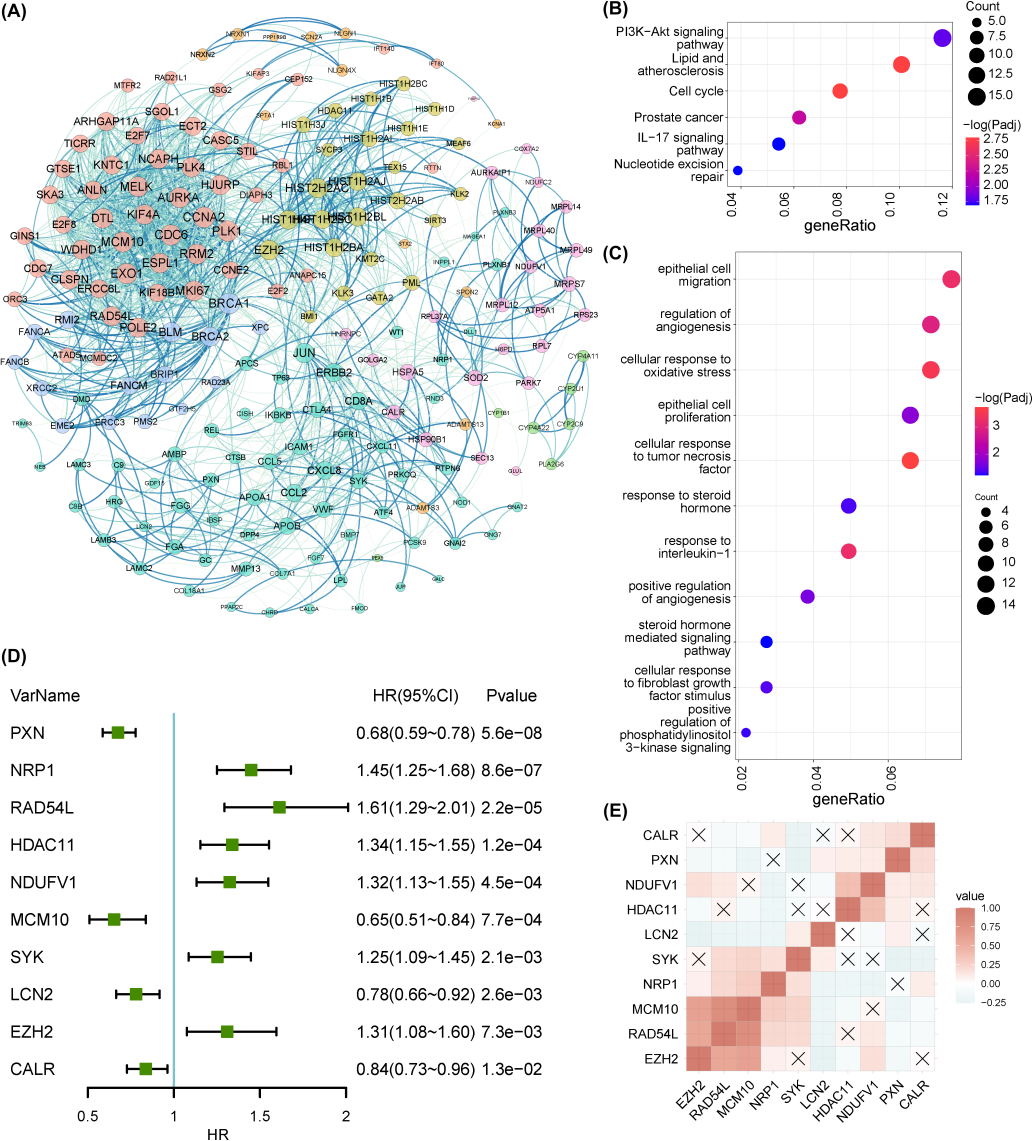
Characteristics of prDEGs and screening of key survival‐related prDEGs. (A) Protein–protein interaction network of prDEGs (progression‐related differentially expressed genes). (B, C) Kyoto Encyclopedia of Genes and Genomes (KEGG) and Gene Ontology (GO) enrichment analysis of top 200 prDEGs. (D) Forest plot of key survival‐related prDEGs revealed by multivariable Cox regression analysis. (E) Correlation matrix of 10 key survival‐related prDEGs.

Utilizing the STRING online database (Version 10.0), we constructed a network of protein–protein interaction (PPI) to discern the hub genes among the prDEGs (Figure 2A). By their degree centrality, the top 200 prDEGs were identified as active participants in the pernicious advancement of PCa. Kyoto Encyclopedia of Genes and Genomes (KEGG) and Gene Ontology (GO) analyses were performed based on these 200 genes. The KEGG enrichment analysis showcased pathways closely associated with progression, including the PI3K‐Akt signalling pathway, cell cycle, interleukin (IL)‐17 signalling pathway, prostate cancer and nucleotide excision repair. In parallel, the GO analysis illuminated pathways on various cellular functions that underlie progression, encompassing cell migration, angiogenesis, energy metabolism, and proliferation. In addition, the enrichment of terms related to the response to steroid hormones and cytokines was observed (Figure 2B,C). These findings collectively shed light on the significant roles played by hub prDEGs in the progression of PCa. Employing multivariate Cox regression analysis coupled with backward elimination, we revealed a total of 10 genes that were related to PFS from the 200 genes. Four of these genes played protective roles, while the remaining six were deemed risk factors (Figure 2D). Additionally, 10 genes showed weak correlations with each other (Figure 2E). These findings substantiate the independent prognostic significance of all 10 genes.

### 3.3 Establishment of a progression‐related risk model in the training set

**FIGURE 3:**
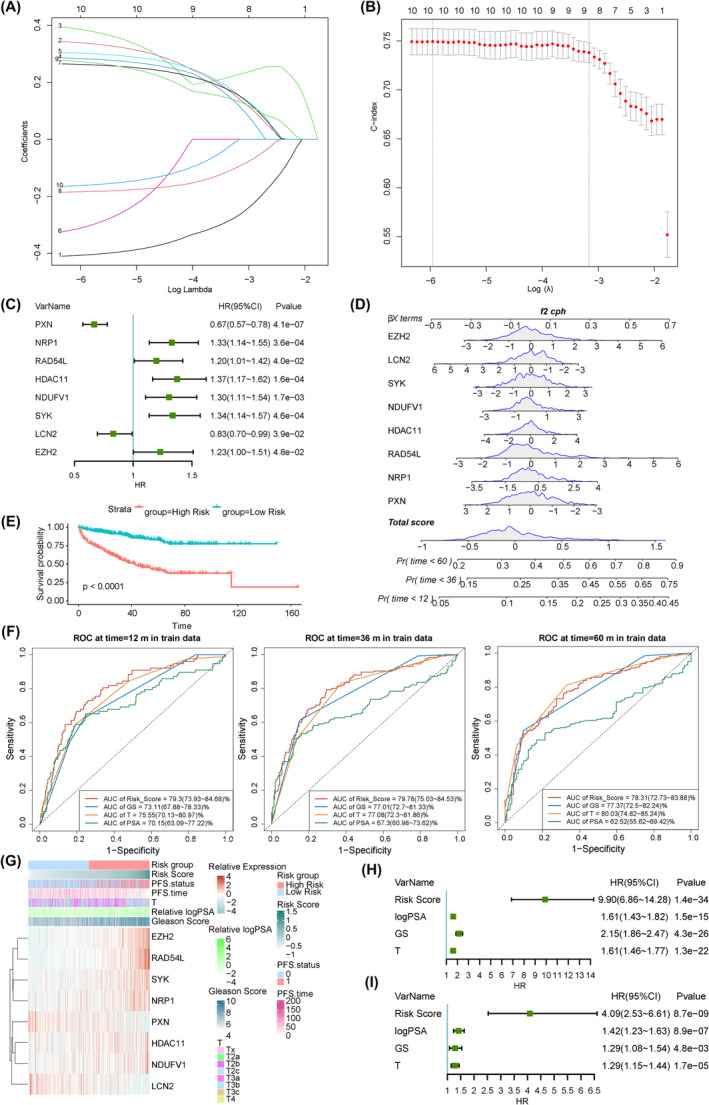
Establishment of a progression‐related risk model in the training set. (A, B) The least absolute shrinkage, lasso and Cox regressions for the PFS‐related key progression‐associated DEGs. (C) The multivariable Cox regression of eight genes further demonstrates the independent prognostic values. (D) A nomogram for predictive PFS probabilities for 1, 3 and 5 years of PCa patients. (E) Kaplan–Meier (KM) analysis for PFS curves of patients from two risk groups. (F) ROC (receiver operating characteristic) analysis of 1‐, 3‐ and 5‐year PFS (progression‐related survival) was applied to evaluate the predictive efficiency. (G) The heatmap of eight‐gene expression profiles, PRS (progression‐related risk score) and clinical indicators in PCa data sets. (H, I) Univariable and multivariable Cox regression analyses of PRS and clinical indicators.

The training set was selected for subsequent analyses. Lasso and Cox regression analyses were employed to evaluate the 10 key survival‐related prDEGs, resulting in the identification of eight genes for further investigation (Figure 3A,B). Utilizing multivariate Cox regression analysis, we calculated the hazard ratio of these eight genes, which demonstrated the independent prognostic value of these genes. As depicted in Figure 3C, NRP1, RAD54L, HDAC11, NDUFV1, SYK and EZH2 were independent risk factors, whereas PXN and LCN2 were independent protective factors. We then performed ridge regression to obtain the coefficients of the eight genes and constructed a nomogram based on these genes to estimate the probabilities of 1‐, 3‐ and 5‐year PFS (Figure S2a,b). Subsequently, the nomogram was employed to calculate the PRS using the following formula: PRS = (−0.11 * z‐score of PXN) + (0.085 * z‐score of NRP1) + (0.12 * z‐score of RAD54L) + (0.058 * z‐score of HDAC11) + (0.080 * z‐score of NDUFV1) + (0.091 * z‐score of SYK) − (0.081 * z‐score of LCN2) + (0.11 * z‐score of EZH2) (Figure 3D). The predictive accuracy of the nomogram was then validated using the bootstrap replicated sample method (Figure S2c). Based on the median PRS, all patients were divided into high‐risk (*n* = 272) and low‐risk (*n* = 273) groups. The results indicated that the high‐risk group exhibited inferior PFS compared to the low‐risk group, with a higher probability of recurrence (Figure 3E). To assess the clinical prognostic potential of this risk model, ROC curves were employed. Remarkably, our model exhibited an AUC of 0.7930, 0.7978 and 0.7831 for 1‐, 3‐ and 5‐year PFS, respectively, surpassing or rivalling other established prognostic indicators for PFS in PCa, such as the Gleason score (GS), T stage (T) and PSA level (Figure 3F). The heatmap in Figure 3G shows the relationship between PRS, clinical indicators and the expression patterns of the eight genes in the training set. Notably, patients in the high‐risk group displayed elevated PSA levels and GSs, as well as shorter PFS. The univariate and multivariate Cox regression results further substantiated the independent and superior prognostic value of our risk score in predicting PFS, surpassing all other clinical indicators (Figure 3H,I).

### 3.4 Validation of the prognostic value of the progression‐related risk model

**FIGURE 4:**
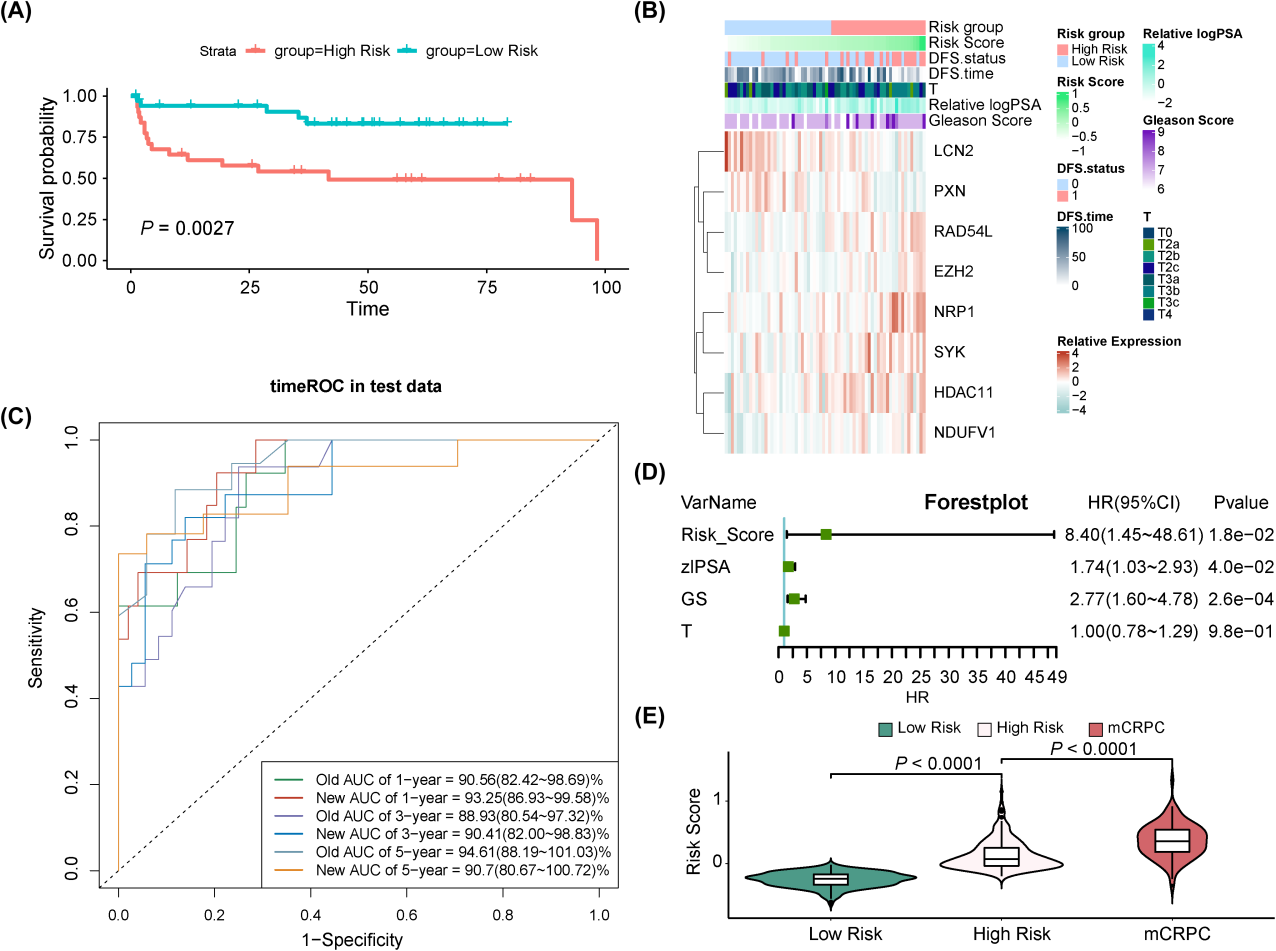
Validation of the prognostic value of the progression‐related risk model and the expression of key prognostic prDEGs in PCa samples. (A) Kaplan–Meier (KM) analysis for PFS curves of patients from the high‐risk group and the low‐risk group in the validation set. (B) The heatmap of eight‐gene expression profiles, PRS and clinical indicators in the validation set. (C) ROC analysis of 1‐, 3‐ and 5‐year PFS to evaluate the predictive efficiency in the validation set. (D) Multivariable Cox regression analysis of PRS and clinical indicators. (E) The risk score of the low‐risk group, high‐risk group and mCRPC group based on the normalized z‐score.

To assess the robustness of our risk model, we calculated the PRS in the validation set using the abovementioned formula. Patients with PCa in the validation set were divided into high‐ and low‐risk groups based on the median PRS. As shown in Figure 4A, individuals in the high‐risk group exhibited shorter PFS, indicative of a poorer prognosis and a heightened likelihood of recurrence. We then validated the relationships between risk scores, clinical indicators and the expression patterns of key genes in the validation set (Figure 4B). The AUC values in the validation set for 1‐, 3‐ and 5‐year PFS were 0.9325, 0.9041 and 0.9070, respectively, underscoring the potential of our risk model as a prognostic tool for PCa. In addition, the ROC curves for 1‐ and 3‐year PFS demonstrated that the prognostic efficiency of PRM surpassed that of other clinical indicators as denoted by the old AUC. However, for 5‐year PFS, the prognostic performance of our risk model was slightly inferior to that of the other indicators (Figure 4C, Figure S2d). We calculated the NRI and IDI between the prognostic value of PRS and PSA in 3‐year PFS, revealing that PRS outperformed PSA (Figure S2e). Furthermore, both univariate and multivariate Cox regression analyses suggested that PRS, PSA, GS and T were independent prognostic clinical indicators (Figure 4D, Figure S2f). By calculating PRS based on combined data sets of patients with localized prostate adenocarcinoma (PRAD) and mCRPC, we compared PRS among the three groups, revealing notably higher PRS levels in mCRPC patients (Figure 4E). In conclusion, these findings provide compelling evidence that our risk model holds considerable predictive value for PFS in PCa.

### 3.5 Characteristics of PRS in single‐cell RNA sequencing data sets

**FIGURE 5:**
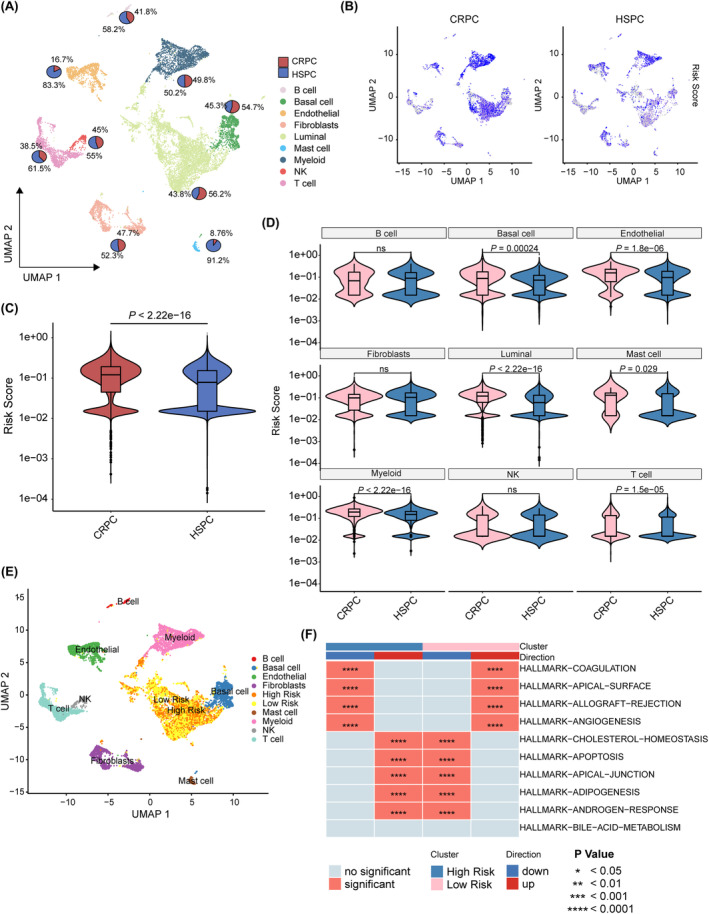
Characteristics of PRS in single‐cell RNA sequencing data sets. (A) Umap plot of distinct cell types and the proportion of cell types in PCa samples. Different colours represent different cell types. (B) Umap plots of risk score distribution of CRPC (left) and HSPC (right). The intensity of colours indicates the risk score of cells. (C) Violin plot of risk score in CRPC and HSPC. (D) Violin plots of risk score of distinct cell types in CRPC and HSPC. (E) Umap plot of risk score distribution of luminal cells. (F) Heatmap illustrating the enrichment scores of the top 10 gene sets in the high PRS group and low PRS group of luminal cells. The red colour represents the significant pathways, and the grey colour represents pathways with no significance. *p < 0.05, **p < 0.01, ***p < 0.001, ****p < 0.0001.

Subsequently, we delved into the detailed distribution of the PRS in CRPC and HSPC using our single‐cell RNA sequencing data sets.^22^ By identifying cell types based on their biomarkers and visualizing them through a umap plot, we depicted the proportion of each cell type in CRPC and HSPC using a pie diagram (Figure 5A and Figure S3a). The PRS of individual cells was calculated using the same nomogram as that used for bulk RNA‐seq in CRPC and HSPC samples. Consistent with the previous findings from bulk RNA sequencing (Figure 4E and 5B,C), we observed that CRPC cells exhibited higher PRS values overall compared to HSPC cells. To further elucidate the differences in PRS across cell types, violin plots were employed, consistently demonstrating significantly higher PRS values in luminal cells, myeloid cells and endothelial cells of CRPC compared to those in HSPC (Figure 5D). Notably, within the luminal cell population, there was significant variation in PRS between CRPC and HSPC. Further analysis specifically focusing on luminal cells revealed that the majority of luminal cells exhibited low PRS, while cells with high PRS were predominantly observed in the CRPC samples (Figure 5E and Figure S3b). Additionally, to explore the relationship between the intracellular heterogeneity of luminal cells and PRS in CRPC and HSPC, we divided luminal cells into high‐risk and low‐risk score groups. Subsequently, we performed irGSEA on CRPC and HSPC, as well as within the high‐risk and low‐risk score groups, to evaluate the differential activation of hallmark pathways in luminal cells. ssGSEA revealed a significant upregulation of the androgen response pathway in the high PRS group compared to the low PRS group (Figure 5F). This finding was consistent when comparing the CRPC group with the HSPC group (Figure S3c). To validate the robustness of our findings, we conducted the same analysis on an independent data set,^25^ which confirmed the consistent upregulation of the androgen response pathway as identified by ssGSEA (Figure S3d). Collectively, these findings suggest a potential association between high PRS and an increased risk of developing CRPC.

### 3.6 SYK serves as a key player in the PRM as well as a tumour promoter in PCa

**FIGURE 6:**
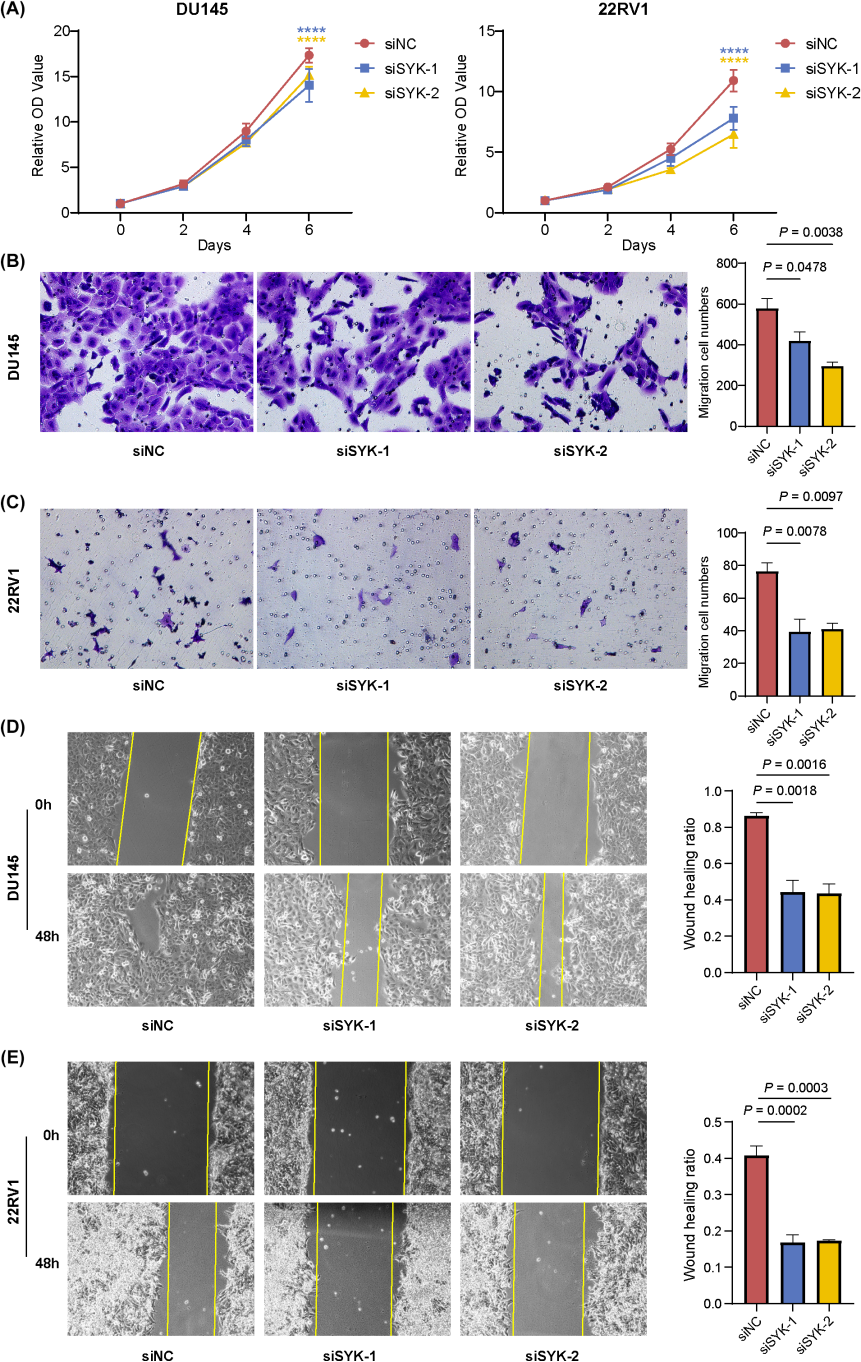
SYK serves as a key player in the PRM as well as a tumour promoter in PCa. (A) SYK silencing inhibited the proliferation of DU145 cells (left) and 22RV1 cells (right). (B, C) The transwell migration assays demonstrating the effects of SYK inhibition on cell migration of DU145 and 22RV1. Quantification of cell migration is shown in the right panel. (D, E) The scratch assays demonstrating the effects of SYK inhibition on cell migration of DU145 and 22RV1. Quantification of cell migration is shown in the right panel. ****p < 0.0001.

To validate the gene expression levels of the independent risk factors in eight key prognostic genes, we conducted RT‐qPCR on 23 paired PCa tissues and adjacent tissues using real samples. As anticipated, NRP1, EZH2, SYK, HDAC11 and NDUFV1 exhibited significant upregulation in tumour tissues, and no significant difference was observed in RAD54L (*p* = 0.054), which aligns with our initial hypothesis (Figure S4a). Furthermore, we performed RT‐qPCR on normal prostate epithelial cells and four PCa cell lines to investigate the altered expression levels of these six genes (Figure S4b). Among the four PCa cell lines, SYK displayed the most pronounced elevation in expression compared to RWPE‐1. Kaplan–Meier survival curves for PFS indicated poorer survival probabilities in patients with high SYK expression in TCGA‐PRAD (Figure S4c). Based on the cumulative evidence, we identified SYK as the key gene associated with the PRS signature and proceeded with additional in vitro molecular biology experiments. To investigate the role of SYK in PCa, we employed siRNA targeting SYK to knock down SYK mRNA levels in 22RV1 and DU145 cells because of the high expression of SYK in the two PCa cells. It was observed that transfection of SYK siRNA significantly decreased SYK expression in 22RV1 and DU145 cells (Figure S4d,e). The cell proliferation assay revealed that knockdown of SYK significantly inhibited the proliferation of DU145 and 22RV1 cells (Figure 6A). Moreover, the downregulation of SYK led to a significant inhibition of migration in PCa cancer cells, as demonstrated by transwell migration assays (Figure 6B,C) and scratch wound healing assays (Figure 6D,E). Up to this point, our data confirmed the key role of SYK in the progression of PCa.

## 4 DISCUSSION

Most patients with PCa generally experience long survival times and favourable prognoses through effective clinical treatments like ADT or radical surgery. Nevertheless, some patients who develop advanced PCa or CRPC face poorer prognoses characterized by recurrence or distinct metastases. Hence, it is necessary to investigate new biomarkers that can predict PCa recurrence. In our study, we identified eight prognostic prDEGs and constructed a PRM based on PFS. This model provides a relatively accurate clinical prognostic tool that can guide treatment decisions for PCa patients.

Herein, we first identified 1454 jointly regulated DEGs and we discovered that these patients could be divided into three subtypes based on these DEGs. The KM curves of the three subtypes showed significant differences in PFS.

Subsequently, a PPI network was constructed, and eight key survival‐related genes were screened to construct a robust risk model. EZH2, RAD54L, SYK, NRP1, HDAC11 and NDUFV1 exhibited close correlations with an unfavourable prognosis, whereas PXN and LCN2 displayed the opposite trend. Lanbo et al. reported that an EZH2 inhibitor enhanced enzalutamide‐induced inhibition of proliferation.^26^ RAD54L, a DNA damage repair gene involved in homologous recombination and DNA repair, was found to promote the progression of CRPC.^27^ SYK was reported to act as a tumour suppressor that inhibits tumorigenesis in breast cancer, while aberrant expression of SYK was found to be upregulated in PCa and related to aggressive progression.^28^, ^29^ NRP1 is associated with PCa progression via the activation of c‐MET signalling.^30^ Tse et al. demonstrated that the expression of NRP1 was upregulated during androgen target therapies and identified NRP1 as a prognostic biomarker of mCRPC.^31^ Wei et al. reported that BCYRN1 promotes proliferation, glucose metabolism and survival of prostate cancer cells by increasing the expression level of HDAC11 in PCa.^32^ The deficiency of NDUFV1 is a common cause of mitochondrial dysfunction.^33^ PXN can act as a regulator in both castration‐sensitive prostate cancer and CRPC.^34^ LCN2 has been identified as a tumour suppressor in colon cancer^35^ and pancreatic cancer,^36^ whereas LCN2 promotes tumorigenesis in PCa.^37^

Utilizing the aforementioned eight genes, we proceeded to calculate the PRS and construct the PRM to provide prognostic predictions for patients. Notably, in both the training and validation sets, patients classified as high‐risk exhibited significantly lower PFS, indicating a heightened likelihood of PCa recurrence. Furthermore, the AUC values for 1‐, 3‐ and 5‐year PFS demonstrated highly favourable outcomes in both the training and validation sets, underscoring the robust and consistent predictive capability of our risk model. Through univariate and multivariate Cox regression analyses, we substantiated that the PRS serves as a precise and superior independent clinical prognostic indicator for PCa. This model holds promising potential as an effective complement to the existing GS system.

Our comprehensive findings provide compelling evidence supporting the pivotal role of SYK dysregulation in the progression of PCa, thereby positioning it as a promising therapeutic target. The observed upregulation of SYK in tumour tissues, coupled with its correlation with inferior PFS, underscores its clinical significance as a prognostic marker. Furthermore, our in vitro experiments reinforce the functional influence of SYK on the proliferation and migration of PCa cells, further substantiating its involvement in the disease process.

Notwithstanding the significant findings of our present study, it is important to acknowledge certain limitations. While our systematic analyses have provided valuable insights into the PRM and its implications for PCa, it is essential to complement these findings with direct experimental evidence in future investigations. Additionally, our results have revealed notable alterations in the molecular characteristics of PCa, necessitating further specific investigations to validate these observed relationships. Furthermore, it is crucial to conduct additional research to ascertain the efficacy of this predictive model for rare pathological subtypes, such as neuroendocrine carcinoma and ductal adenocarcinoma, which constitute a small proportion of cases. Nevertheless, we remain committed to closely monitoring emerging clinical data to further validate the proposed PRM in future endeavours.

## 5 CONCLUSION

In summary, our study developed a novel prognostic risk model utilizing the expression levels of key prDEGs. We validated the accuracy of this model from clinical data to the molecular biology level. Consequently, the PRM holds promise as a valuable adjunctive prognostic tool for predicting the progression of PCa. Furthermore, our exploration of internal relationships using single‐cell RNA sequencing has yielded fresh perspectives on the mechanisms driving PCa progression.

## AUTHOR CONTRIBUTIONS

**Wei Chen:** Conceptualization (lead); project administration (lead); resources (lead); supervision (lead); writing – review and editing (lead). **Yunyan Zhang:** Conceptualization (equal); data curation (lead); formal analysis (lead); methodology (lead); visualization (equal); writing – original draft (lead). **Zhuolin Liu:** Data curation (equal); formal analysis (equal); methodology (equal); visualization (equal); writing – original draft (equal). **Liu Yu:** Data curation (equal); formal analysis (equal); methodology (equal); visualization (supporting); writing – original draft (equal). **Aoyu Fan:** Data curation (supporting); formal analysis (equal); methodology (supporting); visualization (supporting); writing – original draft (supporting). **Yunpeng Li:** Data curation (supporting); formal analysis (supporting); methodology (supporting); visualization (supporting); writing – original draft (supporting). **Xiaobo Li:** Conceptualization (supporting); data curation (supporting); formal analysis (supporting); resources (supporting); writing – review and editing (equal).

## FUNDING INFORMATION

This research did not receive any specific grant from funding agencies in the public, commercial or not‐for‐profit sectors.

## CONFLICT OF INTEREST STATEMENT

The authors declare that they have no competing interests.

## PATIENT CONSENT FOR STATEMENT

All informed consent was obtained from all patients of Zhongshan Hospital, Fudan University.

## Supporting information


Table S1

Table S2

Figures S1–S4

## Data Availability

The data that support the findings of this study are available on request from the corresponding author. The data are not publicly available due to privacy or ethical restrictions. All the R codes used in this study are available from the corresponding author upon request.

## References

[1] Sung H , Ferlay J , Siegel RL , et al. Global cancer statistics 2020: GLOBOCAN estimates of incidence and mortality worldwide for 36 cancers in 185 countries. CA Cancer J Clin. 2021;71:209‐249. 33538338 10.3322/caac.21660

[2] Pound CR , Partin AW , Eisenberger MA , Chan DW , Pearson JD , Walsh PC . Natural history of progression after PSA elevation following radical prostatectomy. JAMA. 1999;281:1591‐1597. 10235151 10.1001/jama.281.17.1591

[3] Roobol MJ , Carlsson SV . Risk stratification in prostate cancer screening, nature reviews. Urology. 2013;10:38‐48. 23247693 10.1038/nrurol.2012.225

[4] Ciccarese C , Massari F , Iacovelli R , et al. Prostate cancer heterogeneity: discovering novel molecular targets for therapy. Cancer Treat Rev. 2017;54:68‐73. 28231559 10.1016/j.ctrv.2017.02.001

[5] Uchio EM , Aslan M , Wells CK , Calderone J , Concato J . Impact of biochemical recurrence in prostate cancer among US veterans. Arch Intern Med. 2010;170:1390‐1395. 20696967 10.1001/archinternmed.2010.262

[6] Crawford ED , Heidenreich A , Lawrentschuk N , et al. Androgen‐targeted therapy in men with prostate cancer: evolving practice and future considerations. Prostate Cancer Prostatic Dis. 2019;22:24‐38. 30131604 10.1038/s41391-018-0079-0 PMC6370592

[7] Ceder Y , Bjartell A , Culig Z , Rubin MA , Tomlins S , Visakorpi T . The molecular evolution of castration‐resistant prostate cancer, European urology. Focus. 2016;2:506‐513. 10.1016/j.euf.2016.11.012 28723516

[8] Rebello RJ , Oing C , Knudsen KE , et al. Prostate cancer. Nat Rev Dis Primers. 2021;7:1‐27. 33542230 10.1038/s41572-020-00243-0

[9] Kirby M , Hirst C , Crawford ED . Characterising the castration‐resistant prostate cancer population: a systematic review. Int J Clin Pract. 2011;65:1180‐1192. 21995694 10.1111/j.1742-1241.2011.02799.x

[10] Hu R , Dunn TA , Wei S , et al. Ligand‐independent androgen receptor variants derived from splicing of cryptic exons signify hormone‐refractory prostate cancer. Cancer Res. 2009;69:16‐22. 19117982 10.1158/0008-5472.CAN-08-2764 PMC2614301

[11] Attard G , Reid AHM , Yap TA , et al. Phase I clinical trial of a selective inhibitor of CYP17, abiraterone acetate, confirms that castration‐resistant prostate cancer commonly remains hormone driven, journal of clinical oncology: official journal of the American society of. Clin Oncol. 2008;26:4563‐4571. 10.1200/JCO.2007.15.9749 18645193

[12] Aakula A , Leivonen S‐K , Hintsanen P , et al. MicroRNA‐135b regulates ERα, AR and HIF1AN and affects breast and prostate cancer cell growth. Mol Oncol. 2015;9:1287‐1300. 25907805 10.1016/j.molonc.2015.03.001 PMC5528813

[13] Mounir M , Lucchetta M , Silva TC , et al. New functionalities in the TCGAbiolinks package for the study and integration of cancer data from GDC and GTEx. PLoS Comput Biol. 2019;15:e1006701. 30835723 10.1371/journal.pcbi.1006701 PMC6420023

[14] Silva TC , Colaprico A , Olsen C , et al. TCGA workflow: analyze cancer genomics and epigenomics data using Bioconductor packages. F1000Res. 2016;5:1542. 28232861 10.12688/f1000research.8923.2 PMC5302158

[15] Li R , Zhu J , Zhong W‐D , Jia Z . PCaDB – a comprehensive and interactive database for transcriptomes from prostate cancer population cohorts. BioRxiv. 2021:2021‐06.

[16] Dobin A , Davis CA , Schlesinger F , et al. STAR: ultrafast universal RNA‐seq aligner. Bioinformatics (Oxford, England). 2013;29:15‐21. 23104886 10.1093/bioinformatics/bts635 PMC3530905

[17] Simon N , Friedman J , Hastie T , Tibshirani R . Regularization paths for Cox's proportional hazards model via coordinate descent. J Stat Softw. 2011;39:1. 10.18637/jss.v039.i05 PMC4824408 27065756

[18] Friedman J , Hastie T , Tibshirani R . Regularization paths for generalized linear models via coordinate descent. J Stat Softw. 2010;33(1):1‐22. 20808728 PMC2929880

[19] Schröder MS , Culhane AC , Quackenbush J , Haibe‐Kains B . Survcomp: an R/Bioconductor package for performance assessment and comparison of survival models. Bioinformatics. 2011;27:3206‐3208. 21903630 10.1093/bioinformatics/btr511 PMC3208391

[20] Haibe‐Kains B , Desmedt C , Sotiriou C , Bontempi G . A comparative study of survival models for breast cancer prognostication based on microarray data: does a single gene beat them all? Bioinformatics. 2008;24:2200‐2208. 18635567 10.1093/bioinformatics/btn374 PMC2553442

[21] Blanche P , Dartigues JF , Jacqmin‐Gadda H . Estimating and comparing time‐dependent areas under receiver operating characteristic curves for censored event times with competing risks. Stat Med. 2013;32:5381‐5397. 24027076 10.1002/sim.5958

[22] Zhang Y , Fan A , Li Y , et al. Single‐cell RNA sequencing reveals that HSD17B2 in cancer‐associated fibroblasts promotes the development and progression of castration‐resistant prostate cancer. Cancer Lett. 2023;566:216244. 37244445 10.1016/j.canlet.2023.216244

[23] Xu D , Ma R , Ju Y , et al. Cholesterol sulfate alleviates ulcerative colitis by promoting cholesterol biosynthesis in colonic epithelial cells. Nat Commun. 2022;13:4428. 35908039 10.1038/s41467-022-32158-7 PMC9338998

[24] Gaujoux R , Seoighe C . A flexible R package for nonnegative matrix factorization. BMC Bioinformatics. 2010;11:367. 20598126 10.1186/1471-2105-11-367 PMC2912887

[25] Chen S , Zhu G , Yang Y , et al. Single‐cell analysis reveals transcriptomic remodellings in distinct cell types that contribute to human prostate cancer progression. Nat Cell Biol. 2021;23:87‐98. 33420488 10.1038/s41556-020-00613-6

[26] Xiao L , Tien JC , Vo J , et al. Epigenetic reprogramming with antisense oligonucleotides enhances the effectiveness of androgen receptor inhibition in castration‐resistant prostate cancer. Cancer Res. 2018;78:5731‐5740. 30135193 10.1158/0008-5472.CAN-18-0941 PMC6191320

[27] Li L , Chang W , Yang G , et al. Targeting poly (ADP‐ribose) polymerase and the c‐Myb‐TopBP1‐ATR‐Chk1 signaling pathway in castration‐resistant prostate cancer. Sci Signal. 2014;7:ra47. 24847116 10.1126/scisignal.2005070 PMC4135429

[28] Coopman PJP , Do MTH , Barth M , et al. The Syk tyrosine kinase suppresses malignant growth of human breast cancer cells. Nature. 2000;406:742‐747. 10963601 10.1038/35021086

[29] Ghotra VPS , He S , van der Horst G , et al. SYK is a candidate kinase target for the treatment of advanced prostate cancer. Cancer Res. 2015;75:230‐240. 25388286 10.1158/0008-5472.CAN-14-0629

[30] Zhang S , Zhau HE , Osunkoya AO , et al. Vascular endothelial growth factor regulates myeloid cell leukemia‐1 expression through neuropilin‐1‐dependent activation of c‐MET signaling in human prostate cancer cells. Mol Cancer. 2010;9:9. 20085644 10.1186/1476-4598-9-9 PMC2820018

[31] Tse BWC , Volpert M , Ratther E , et al. Neuropilin‐1 is upregulated in the adaptive response of prostate tumors to androgen‐targeted therapies and is prognostic of metastatic progression and patient mortality. Oncogene. 2017;36:3417‐3427. 28092670 10.1038/onc.2016.482 PMC5485179

[32] Huo W , Qi F , Wang K . Long non‐coding RNA BCYRN1 promotes prostate cancer progression via elevation of HDAC11. Oncol Rep. 2020;44:1233‐1245. 32705287 10.3892/or.2020.7680

[33] Varghese F , Atcheson E , Bridges HR , Hirst J . Characterization of clinically identified mutations in NDUFV1, the flavin‐binding subunit of respiratory complex I, using a yeast model system. Hum Mol Genet. 2015;24:6350‐6360. 26345448 10.1093/hmg/ddv344 PMC4614703

[34] Sen A , De Castro I , DeFranco DB , et al. Paxillin mediates extranuclear and intranuclear signaling in prostate cancer proliferation. J Clin Invest. 2012;122:2469‐2481. 22684108 10.1172/JCI62044 PMC3386821

[35] Chaudhary N , Choudhary BS , Shah SG , et al. Lipocalin 2 expression promotes tumor progression and therapy resistance by inhibiting ferroptosis in colorectal cancer. Int J Cancer. 2021;149:1495‐1511. 34146401 10.1002/ijc.33711

[36] Tong Z , Kunnumakkara AB , Wang H , et al. Neutrophil gelatinase–associated lipocalin: a novel suppressor of invasion and angiogenesis in pancreatic cancer. Cancer Res. 2008;68(15):6100‐6108. 18676832 10.1158/0008-5472.CAN-08-0540 PMC2714276

[37] Schröder SK , Pinoé‐Schmidt M , Weiskirchen R . Lipocalin‐2 (LCN2) deficiency leads to cellular changes in highly metastatic human prostate cancer cell line PC‐3. Cells. 2022;11:260. 35053376 10.3390/cells11020260 PMC8773519

