# Supplementary material for: Integrative bioinformatics approach yields a novel gene expression risk model for prognosis and progression prediction in prostate cancer
Source: J Cell Mol Med. 2024 Jun 6;28(11):e18405. doi: 10.1111/jcmm.18405 (PMC11154836; doi:10.1111/jcmm.18405)
Supplement: Supplementary file 1 — Table S1 [file JCMM-28-e18405-s003.docx]

Table S1. Primer sequences of EZH2, SYK, HDAC11, NRP1, RAD54L, and NDUFV1 genes.

| Gene | Forward: 5’-3’ | Revise: 5’-3’ |
| --- | --- | --- |
| EZH2 | CCAAGGAAAAAGAAGAGGAAACACC | GCTATCACACAAGGGCACGA |
| SYK | TTGGTCAGCGGGTGGAATAA | GCAGGGGAGGACTTTCTGTG |
| HDAC11 | TGTTTGAGCGTGTGGAGGG | CTGGGTAGATGTGGCGGTTG |
| NRP1 | ACGGGAACTTGGTGGATGAA | AGGTCTTGTGAGAGCCCCAG |
| RAD54L | CCTATGAGACCTTCCGCCTTC | TGACCAGACCAACACTTCCT |
| NDUFV1 | CGGGTATCTGTGCGTTTCAG | TTCAGCGAGCCAAATGAGGT |
