# Supplementary material for: Integrative bioinformatics approach yields a novel gene expression risk model for prognosis and progression prediction in prostate cancer
Source: J Cell Mol Med. 2024 Jun 6;28(11):e18405. doi: 10.1111/jcmm.18405 (PMC11154836; doi:10.1111/jcmm.18405)
Supplement: Supplementary file 3 — Figures S1–S4 [file JCMM-28-e18405-s001.docx]

**Supplementary Figure 1**


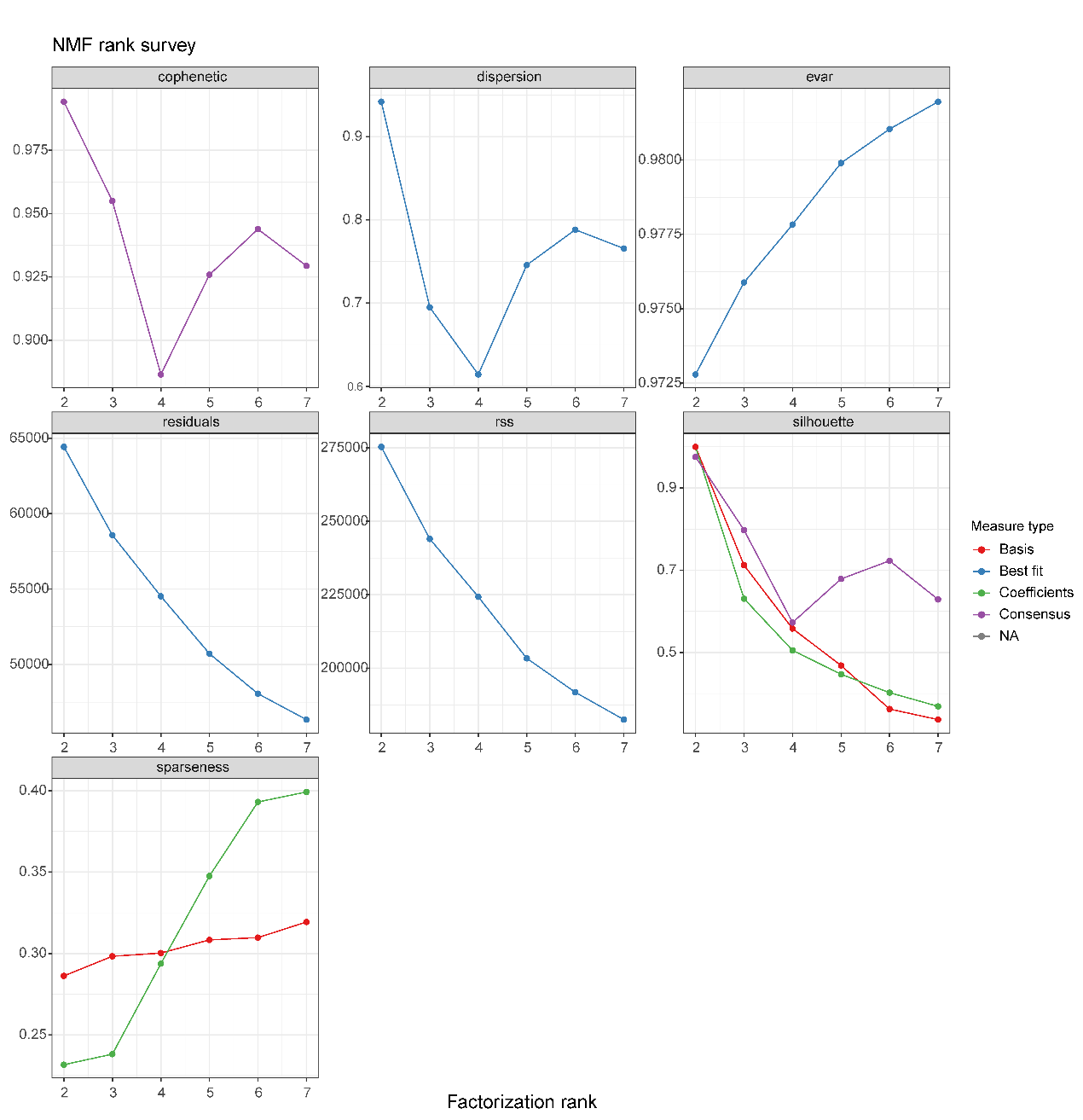


**Supplementary Figure 1** NMF rank survey for the selection of an appropriate k value.

**Supplementary Figure 2**


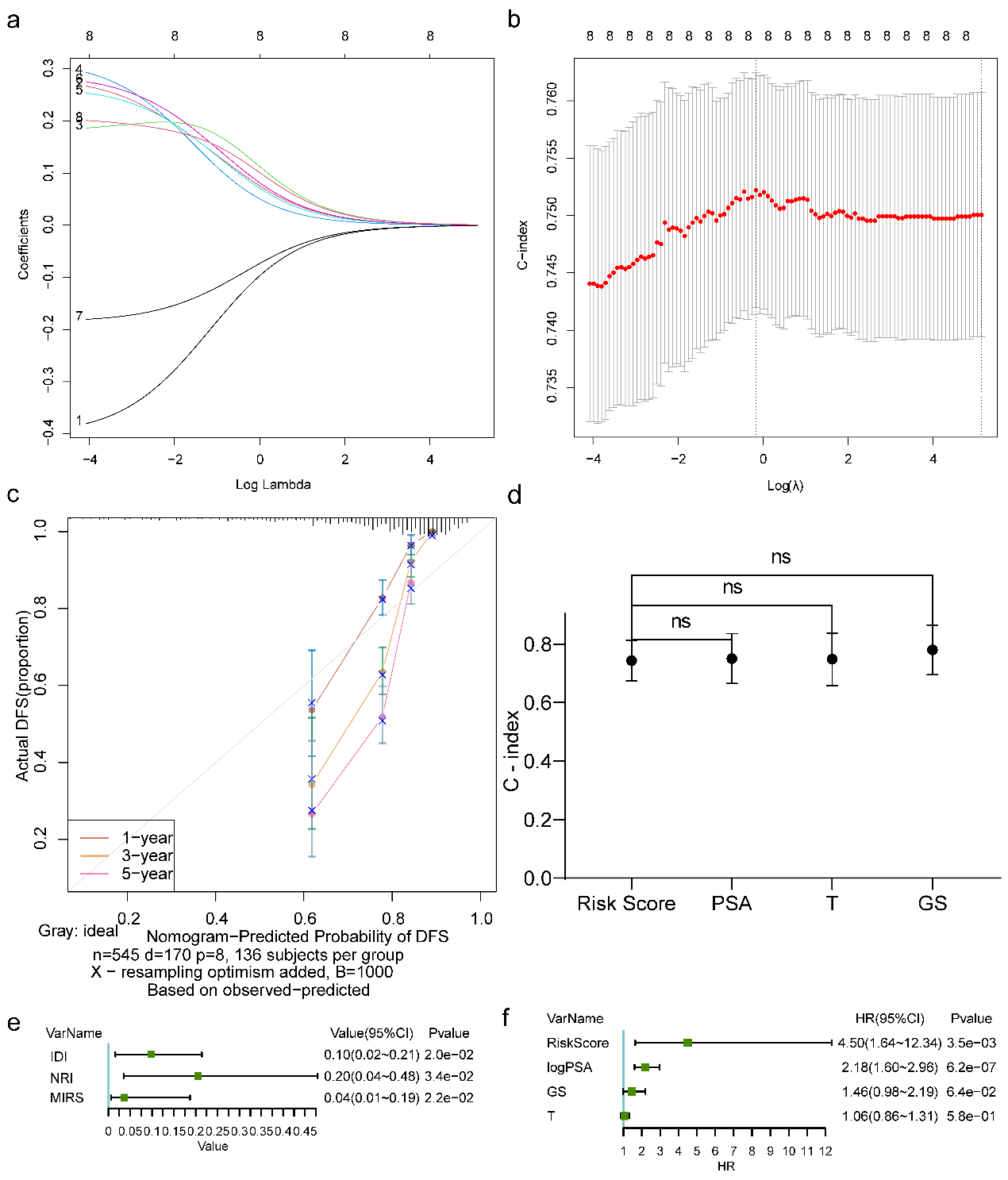


**Supplementary Figure 2** **a, b** Ridge regression for the PFS-related key recurrence-associated DEGs. **c** Calibration curve of a nomogram to confirm the accuracy. **d** C-index of risk score and classic clinical indicators. **e** The plot of IDI and NRI. **f** Univariable Cox regression analysis of PRS and clinical indicators.

**Supplementary Figure 3**


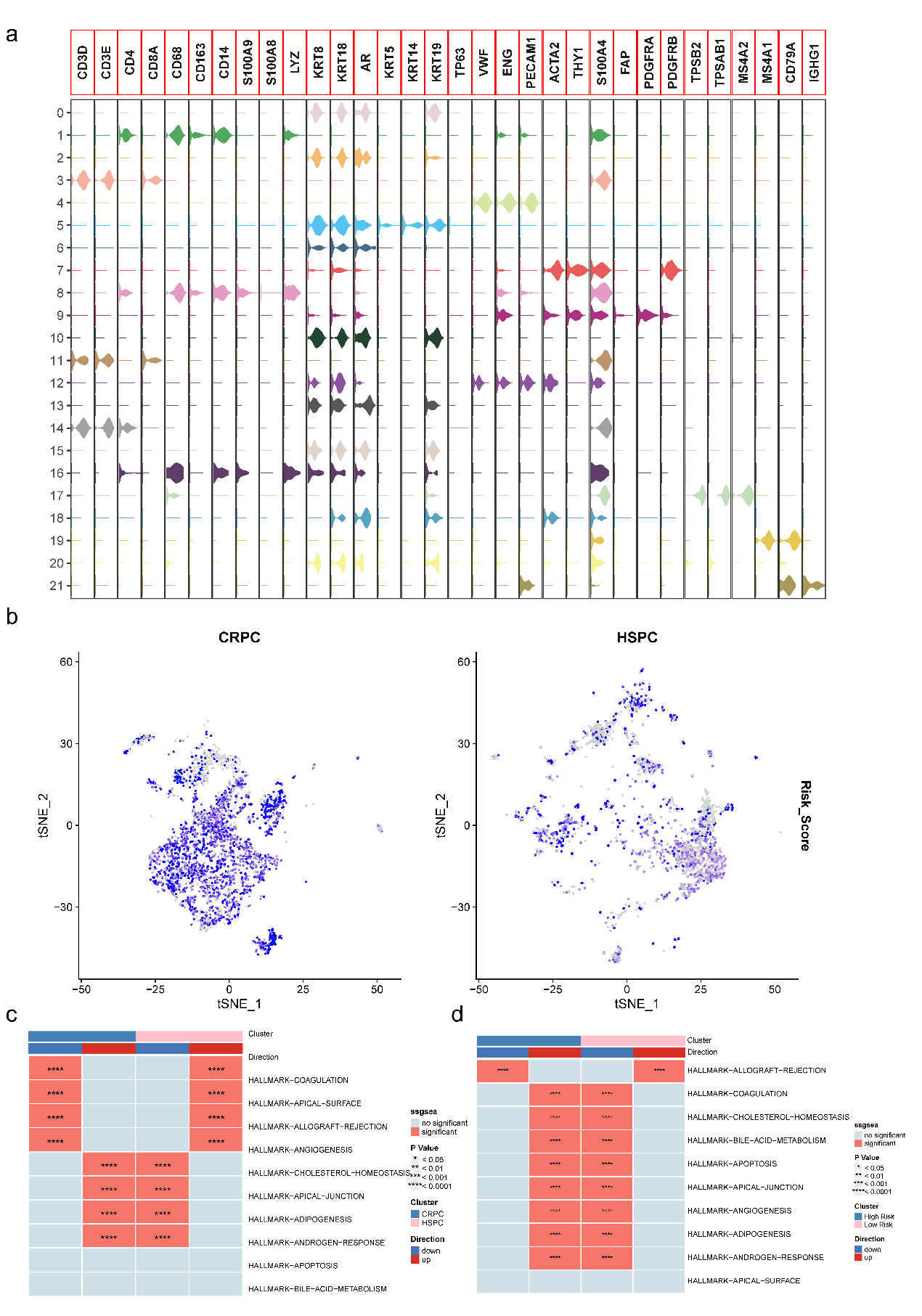


**Supplementary Figure 3** Details of the single-cell RNA-seq analysis. **a** Violin plots of cell markers for 22 clusters. **b** UMAP plot of risk score distribution in epithelial subtypes of CRPC (left) and HSPC (right). **c** Heatmap illustrating the enrichment scores of the top 10 gene sets in luminal cells of CRPC and HSPC. The red color represents the significant pathways, and the gray color represents pathways with no significance. *P < 0.05, **P < 0.01, ***P < 0.001, ****P < 0.0001. **d** Heatmap illustrating the enrichment scores of the top 10 gene sets in luminal cells of high risk group and low risk group in the public datasets. The red color represents the significant pathways, and the gray color represents pathways with no significance. *P < 0.05, **P < 0.01, ***P < 0.001, ****P < 0.0001.

**Supplementary Figure 4**


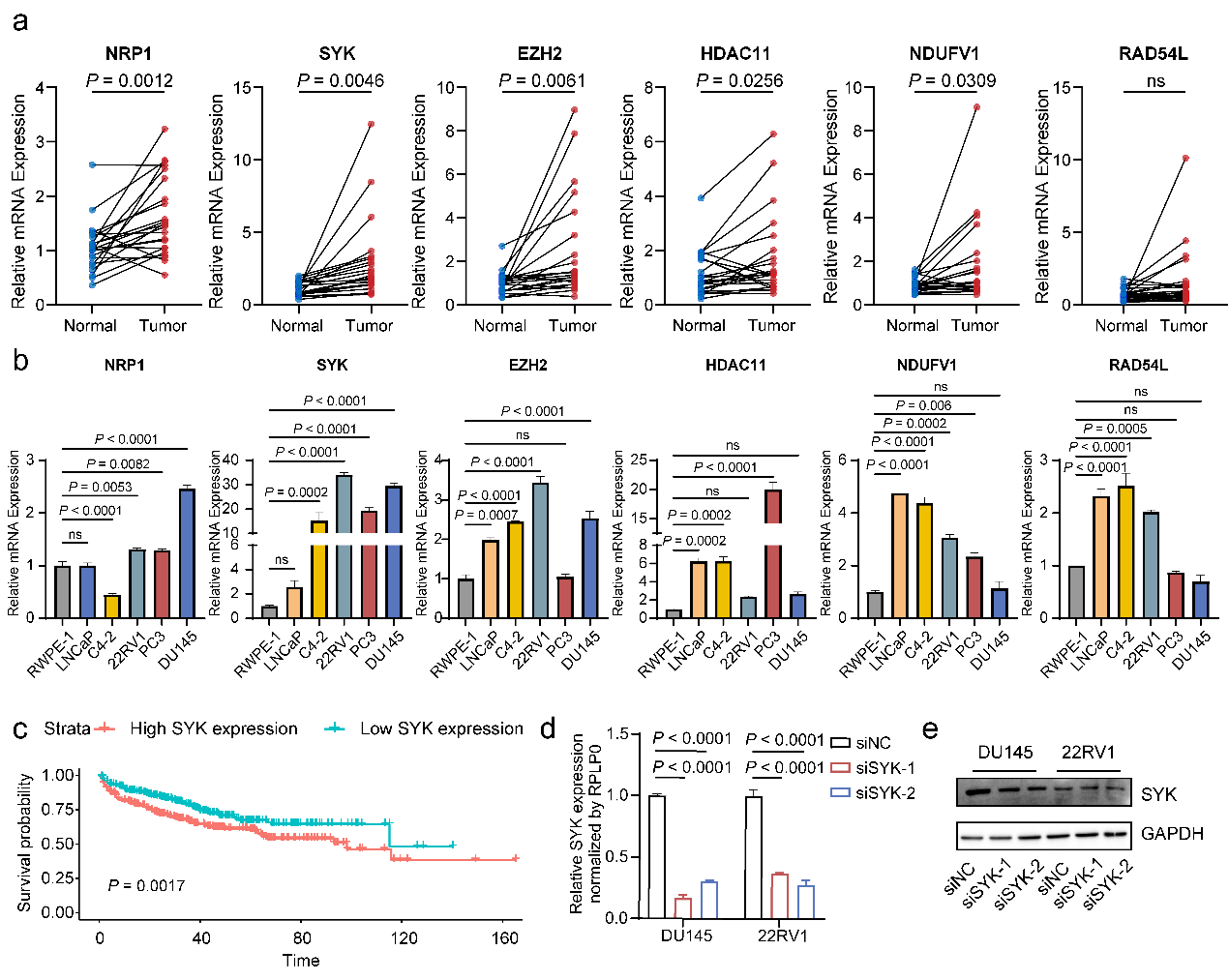


**Supplementary Figure 4** SYK serves as a key player in the PRM as well as a tumor promoter in PCa. **a** Expression levels of key prognostic prDEGs in PCa tissues and their matched adjacent tissues normalized to β-actin (n=23). **b** The relative expression levels of each gene in cell lines normalized to β-actin. **c** The KM survival plot illustrates the impact of SYK expression on PFS in PCa. **d** Detection of SYK mRNA knockdown in DU145 and 22RV1 cells by RT-qPCR. **e** Detection of SYK mRNA knockdown in DU145 and 22RV1 cells by western blotting. GAPDH serves as loading control.
